# Global estimates on the number of people blind or visually impaired by glaucoma: A meta-analysis from 2000 to 2020

**DOI:** 10.1038/s41433-024-02995-5

**Published:** 2024-04-02

**Authors:** Vision Loss Expert Group of the Global Burden of Disease Study, Rupert R. A. Bourne, Jost B. Jonas, David Friedman, Vinay Nangia, Alain Bron, Ian Tapply, Arthur G. Fernandes, Maria Vittoria Cicinelli, Alessandro Arrigo, Nicolas Leveziel, Serge Resnikoff, Hugh R. Taylor, Tabassom Sedighi, Mukkharram M. Bikbov, Tasanee Braithwaite, Ching-Yu Cheng, Nathan Congdon, Monte A. Del Monte, Joshua R. Ehrlich, Tim Fricke, João M. Furtado, Gus Gazzard, Ronnie George, M. Elizabeth Hartnett, Rim Kahloun, John H. Kempen, Moncef Khairallah, Rohit C. Khanna, Judy E. Kim, Van Charles Lansingh, Janet Leasher, Kovin S. Naidoo, Michal Nowak, Konrad Pesudovs, Tunde Peto, Pradeep Ramulu, Fotis Topouzis, Mitiadis Tsilimbaris, Ya Xing Wang, Ningli Wang, Seth Flaxman, the GBD 2019 Blindness and Vision Impairment Collaborators, Rupert R. A. Bourne, Jost B. Jonas, Robert James Casson, David S. Friedman, Vinay Nangia, Alain M. Bron, Ian Tapply, Arthur G. Fernandes, Maria Vittoria Cicinelli, Nicolas Leveziel, Paul Svitil Briant, Theo Vos, Serge Resnikoff, Yohannes Habtegiorgis Abate, Melsew Dagne Abate, Zahra Abbasi Dolatabadi, Mozhan Abdollahi, Richard Gyan Aboagye, Eman Abu-Gharbieh, Salahdein Aburuz, Qorinah Estiningtyas Sakilah Adnani, Shahin Aghamiri, Bright Opoku Ahinkorah, Danish Ahmad, Hamid Ahmadieh, Hooman Ahmadzadeh, Ayman Ahmed, Ahmad Samir Alfaar, Cyrus Alinia, Louay Almidani, Hubert Amu, Sofia Androudi, Abhishek Anil, Jalal Arabloo, Damelash Areda, Tahira Ashraf, Sara Bagherieh, Ovidiu Constantin Baltatu, Mehmet Firat Baran, Amadou Barrow, Azadeh Bashiri, Nebiyou Simegnew Bayileyegn, Fatemeh Bazvand, Alemshet Yirga Berhie, Jasvinder Singh Bhatti, Mukharram Bikbov, Marina G. Birck, Veera R. Bitra, Marija M. Bozic, Tasanee Braithwaite, Katrin Burkart, Yasser Bustanji, Zahid A. Butt, Muthia Cenderadewi, Vijay Kumar Chattu, Kaleb Coberly, Omid Dadras, Xiaochen Dai, Ana Maria Dascalu, Anna Dastiridou, Vinoth Gnana Chellaiyan Devanbu, Meghnath Dhimal, Daniel Diaz, Thao Huynh Phuong Do, Thanh Chi Do, Arkadiusz Marian Dziedzic, Joshua R. Ehrlich, Michael Ekholuenetale, Muhammed Elhadi, Mohammad Hassan Emamian, Mehdi Emamverdi, Hossein Farrokhpour, Getahun Fetensa, Florian Fischer, Ali Forouhari, Kayode Raphael Fowobaje, João M. Furtado, Aravind P. Gandhi, Miglas W. W. Gebregergis, Bárbara Niegia Garcia Goulart, Mesay Dechasa Gudeta, Sapna Gupta, Vivek Kumar Gupta, Veer Bala Gupta, Golnaz Heidari, Sung Hwi Hong, Hong-Han Huynh, Segun Emmanuel Ibitoye, Irena M. Ilic, Mustapha Immurana, Sathish Kumar Jayapal, Nitin Joseph, Charity Ehimwenma Joshua, Rim Kahloun, Himal Kandel, Ibraheem M. Karaye, Hengameh Kasraei, Getu Mosisa Kebebew, John H. Kempen, Mahmoud Tawfik KhalafAlla, Sudarshan Khanal, Mahalaqua Nazli Khatib, Kewal Krishan, Chandrakant Lahariya, Janet L. Leasher, Stephen S. Lim, Roy Rillera Marzo, Andrea Maugeri, Yang Meng, Tomislav Mestrovic, Manish Mishra, Nouh Saad Mohamed, Hoda Mojiri-forushani, Ali H. Mokdad, Hamed Momeni-Moghaddam, Fateme Montazeri, Admir Mulita, Christopher J. L. Murray, Mahdi Nabi Foodani, Ganesh R. Naik, Zuhair S. Natto, Biswa Prakash Nayak, Mohammad Negaresh, Hadush Negash, Dang H. Nguyen, Bogdan Oancea, Andrew T. Olagunju, Matthew Idowu Olatubi, Wael M. S. Osman, Uchechukwu Levi Osuagwu, Jagadish Rao Padubidri, Songhomitra Panda-Jonas, Shahina Pardhan, Seoyeon Park, Jay Patel, Arokiasamy Perianayagam, Konrad Pesudovs, Hoang Tran Pham, Elton Junio Sady Prates, Ibrahim Qattea, Fakher Rahim, Mosiur Rahman, Deepthi Rapaka, Salman Rawaf, Nazila Rezaei, Priyanka Roy, Basema Saddik, Umar Saeed, Sher Zaman Safi, Sare Safi, Joseph W. Sakshaug, Mohamed A. Saleh, Vijaya Paul Samuel, Abdallah M. Samy, Aswini Saravanan, Allen Seylani, Masood Ali Shaikh, Muhammad Aaqib Shamim, Mohammed Shannawaz, Bereket Beyene Shashamo, Maryam Shayan, Aminu Shittu, Emmanuel Edwar Siddig, Jasvinder A. Singh, Yonatan Solomon, Raúl A. R. C. Sousa, Seyyed Mohammad Tabatabaei, Mohammad Tabish, Jansje Henny Vera Ticoalu, Temesgen Mohammed Toma, Aristidis Tsatsakis, Guesh Mebrahtom Tsegay, Rohollah Valizadeh, Maria Viskadourou, Gizachew Tadesse Wassie, Nuwan Darshana Wickramasinghe, Dong Keon Yon, Yuyi You, Seth Flaxman, Jaimie D. Steinmetz

**Affiliations:** 1 Vision and Eye Research Institute, Anglia Ruskin University, Cambridge, UK; 2 Department of Ophthalmology, Medical Faculty Mannheim, Heidelberg University, Heidelberg, Germany; 3 Massachusetts Eye and Ear, Harvard Medical School, Boston, US; 4 Suraj Eye Institute, 559 New Colony, Nagpur, India; 5 University Hospital, Dijon, France; 6 Addenbrooke’s Hospital, Cambridge, UK; 7 Federal University of Sao Paolo, Sao Paolo, SP Brazil; 8 University of Calgary, Calgary, AB Canada; 9 School of Medicine, Vita-Salute San Raffaele University, Milan, Italy; 10 Department of Ophthalmology, IRCCS San Raffaele Scientific Institute, Milan, Italy; 11 Scientific Institute San Raffaele Hospital, Vita-Salute University, Milan, Italy; 12 University of Poitiers, Poitiers, France; 13 CHU de Poitiers, Poitiers, France; 14 Brien Holden Vision Institute, Sydney, NSW Australia; 15 School of Optometry and Vision Sciences, Faculty of Medicine, University of New South Wales, Sydney, NSW Australia; 16 School of Population and Global Health, University of Melbourne, Carlton, VIC Australia; 17 Ufa Eye Research Institute, Ufa, Russia; 18 School of Life Course and Population Sciences, King’s College London, London, UK; 19 The Medical Eye Unit, Guy’s and St Thomas’ NHS Foundation Trust, London, UK; 20 National University of Singapore, Singapore, Singapore; 21 Singapore Eye Research Institute, Singapore, Singapore; 22 Queen’s University Belfast, Belfast, Northern Ireland UK; 23 Orbis International, New York, USA; 24 Zhongshan Ophthalmic Center, Sun Yat-sen University, Guangzhou, China; 25 University of Michigan, Ann Arbor, USA; 26 Kellogg Eye Center, Ann Arbor, USA; 27 Institute for Social Research, University of Michigan, Ann Arbor, USA; 28 Department of Ophthalmology and Visual Sciences, University of Michigan, Ann Arbor, USA; 29 Australian College of Optometry, Vic, Australia; 30 University of Melbourne, Vic, Australia; 31 UNSW Sydney, Sydney, NSW Australia; 32 Ribeirão Preto Medical School, University of São Paulo, São Paulo, Brazil; 33 Institute of Ophthalmology UCL & NIHR Biomedical Research Centre, London, UK; 34 Sankara Nethralaya, Medical Research Foundation, Chennai, India; 35 Stanford University, Stanford, USA; 36 Associated Ophthalmologists of Monastir, Monastir, Tunisia; 37 Department of Ophthalmology, Harvard University, Boston, MA USA; 38 Eye Unit, MyungSung Medical College, Addis Ababa, Ethiopia; 39 Department of Ophthalmology, Addis Ababa University, Addis Ababa, Ethiopia; 40 Sight for Souls, Bellevue, WA USA; 41 Fattouma Bourguiba University Hospital, University of Monastir, Monastir, Tunisia; 42 Allen Foster Community Eye Health Research Centre, Gullapalli Pratibha Rao International Centre for Advancement of Rural Eye care, L.V. Prasad Eye Institute, Hyderabad, India; 43 Brien Holden Eye Research Centre, L.V. Prasad Eye Institute, Banjara Hills, Hyderabad, India; 44 School of Optometry and Vision Science, University of New South Wales, Sydney, Australia; 45 University of Rochester, School of Medicine and Dentistry, Rochester, NY USA; 46 University of Texas Southwestern Medical Center, Dallas, US; 47 HelpMeSee, Instituto Mexicano de Oftalmologia, Santiago de Querétaro, Mexico; 48 University of Miami, Coral Gables, US; 49 University of Utah, Salt Lake City, US; 50 Nova Southeastern University College for Optometry, Fort Lauderdale, Florida USA; 51 African Vision Research Institute, University of KwaZulu-Natal (UKZN), Berea, South Africa; 52 School of Optometry and Vision Science, University of New South Wales, Kensington, Australia; 53 Institute of Optics and Optometry, University of Social Science, Lodz, Poland; 54 Medicine & Health, University of New South Wales, Kensington, Australia; 55 Centre for Public Health, Queens University Belfast, Belfast, Northern Ireland UK; 56 John Hopkins Wilmer Eye Institute, Baltimore, US; 57 st Department of Ophthalmology, Medical School, Aristotle University of Thessaloniki, Ahepa Hospital, Thessaloniki, Greece; 58 University of Crete Medical School, Giofirakia, Greece; 59 Beijing Institute of Ophthamology, Beijing Tongren Hospital, Capital Medical University, Beijing Ophthamology and Visual Sciences Key Laboratory, Beijing, China; 60 Beijing Institute of Ophthamology, Beijing Tongren Eye Center, Beijing Tongren Hospital, Capital Medical University, Beijing, China; 61 Department of Computer Science, University of Oxford, Oxford, UK; 62 Institute of Molecular and Clinical Ophthalmology Basel, Basel, Switzerland; 63 Department of Ophthalmology, Heidelberg University, Mannheim, Germany; 64 Department of Ophthalmology, University of Adelaide, Adelaide, SA Australia; 65 Mass Eye and Ear Department of Ophthalmology, Harvard Medical School, Boston, MA USA; 66 Suraj Eye Institute, Nagpur, India; 67 Department of Ophthalmology, University Hospital Dijon, Dijon, France; 68 Eye and Nutrition Research Group, Université Bourgogne Franche-Comté, Dijon, France; 69 Department of Ophthalmology, Cambridge University Hospitals, Cambridge, UK; 70 Department of Ophthalmology and Visual Sciences, Federal University of São Paulo, São Paulo, Brazil; 71 Department of Ophthalmology, San Raffaele Scientific Institute, Milan, Italy; 72 Ophthalmology Department, CHU de Poitiers (Poitiers University Hospital), Poitiers, France; 73 Unité 1084, National Institute of Health and Medical Research (INSERM), Poitiers, France; 74 Institute for Health Metrics and Evaluation, University of Washington, Seattle, WA USA; 75 Department of Health Metrics Sciences, School of Medicine, University of Washington, Seattle, WA USA; 76 School of Optometry and Vision Science, University of New South Wales, Sydney, NSW Australia; 77 Department of Clinical Governance and Quality Improvement, Aleta Wondo Hospital, Aleta Wondo, Ethiopia; 78 Department of Nursing, Woldia University, Woldia, Ethiopia; 79 Department of Medical-surgical Nursing, Tehran University of Medical Sciences, Tehran, Iran; 80 School of Medicine, Shiraz University of Medical Sciences, Shiraz, Iran; 81 Department of Family and Community Health, University of Health and Allied Sciences, Ho, Ghana; 82 Clinical Sciences Department, University of Sharjah, Sharjah, United Arab Emirates; 83 Department of Therapeutics, United Arab Emirates University, Al Ain, United Arab Emirates; 84 College of Pharmacy, University of Jordan, Amman, Jordan; 85 Faculty of Medicine, Universitas Padjadjaran (Padjadjaran University), Bandung, Indonesia; 86 Department of Biotechnology, Shahid Beheshti University of Medical Sciences, Tehran, Iran; 87 School of Public Health, University of Technology Sydney, Sydney, NSW Australia; 88 School of Medicine and Psychology, Australian National University, Canberra, ACT Australia; 89 Public Health Foundation of India, Gandhinagar, India; 90 Ophthalmic Research Center, Shahid Beheshti University of Medical Sciences, Tehran, Iran; 91 Department of Ophthalmology, Shahid Beheshti University of Medical Sciences, Tehran, Iran; 92 Bascom Palmer Eye Institute, University of Miami, Miami, FL USA; 93 Institute of Endemic Diseases, University of Khartoum, Khartoum, Sudan; 94 Swiss Tropical and Public Health Institute, University of Basel, Basel, Switzerland; 95 Department of Ophthalmology, University of Leipzig Medical Center, Leipzig, Germany; 96 Department of Ophthalmology, Charité Medical University Berlin, Berlin, Germany; 97 Department of Health Care Management and Economics, Urmia University of Medical Sciences, Urmia, Iran; 98 Wilmer Eye Institute, Johns Hopkins University School of Medicine, Baltimore, MD USA; 99 Doheny Image Reading and Research Lab (DIRRL) Doheny Eye Institute, University of California Los Angeles, Los Angeles, CA USA; 100 Department of Population and Behavioural Sciences, University of Health and Allied Sciences, Ho, Ghana; 101 Department of Medicine, University of Thessaly, Volos, Greece; 102 Department of Pharmacology, All India Institute of Medical Sciences, Jodhpur, India; 103 All India Institute of Medical Sciences, Bhubaneswar, India; 104 Health Management and Economics Research Center, Iran University of Medical Sciences, Tehran, Iran; 105 College of Art and Science, Ottawa University, Surprise, AZ USA; 106 School of Life Sciences, Arizona State University, Tempe, AZ USA; 107 University Institute of Radiological Sciences and Medical Imaging Technology, The University of Lahore, Lahore, Pakistan; 108 School of Medicine, Isfahan University of Medical Sciences, Isfahan, Iran; 109 Center of Innovation, Technology and Education (CITE), Anhembi Morumbi University, Sao Jose dos Campos, Brazil; 110 Vocational School of Technical Sciences, Batman University, Batman, Türkiye; 111 Department of Epidemiology, University of Florida, Gainesville, FL USA; 112 Department of Public & Environmental Health, University of The Gambia, Brikama, The Gambia; 113 Health Information Management, Shiraz University of Medical Sciences, Shiraz, Iran; 114 Department of Surgery, Jimma University, Jimma, Ethiopia; 115 Farabi Eye Hospital, Tehran University of Medical Sciences, Tehran, Iran; 116 School of Health Science, Bahir Dar University, Bahir Dar, Ethiopia; 117 Human Genetics and Molecular Medicine, Central University of Punjab, Bathinda, India; 118 Epidemiology Department, Ufa Eye Research Institute, Ufa, Russia; 119 Division of Clinical Epidemiology, McGill University, Montreal, QC Canada; 120 Centre for Outcomes Research and Evaluation, Research Institute of the McGill University Health Centre, Montreal, QC Canada; 121 Faculty of Health Sciences, University of Botswana, Gaborone, Botswana; 122 Faculty of Medicine, University of Belgrade, Belgrade, Serbia; 123 University Eye Hospital, Clinical Center of Serbia, Belgrade, Serbia; 124 Ophthalmology Department, Moorfields Eye Hospital NHS Foundation Trust, London, UK; 125 International Centre for Eye Health, London School of Hygiene & Tropical Medicine, London, UK; 126 Department of Biopharmaceutics and Clinical Pharmacy, The University of Jordan, Amman, Jordan; 127 Department of Basic Biomedical Sciences, University of Sharjah, Sharjah, United Arab Emirates; 128 School of Public Health and Health Systems, University of Waterloo, Waterloo, ON Canada; 129 Al Shifa School of Public Health, Al Shifa Trust Eye Hospital, Rawalpindi, Pakistan; 130 College of Public Health, Medical and Veterinary Sciences, James Cook University, Townsville, QLD Australia; 131 Public Health Departement, University of Mataram, Mataram, Indonesia; 132 Temerty Faculty of Medicine, University of Toronto, Toronto, ON Canada; 133 Saveetha Dental College, Saveetha University, Chennai, India; 134 Department of Addiction Medicine, Haukland University Hospital, Bergen, Norway; 135 Department of Global Public Health and Primary Care, University of Bergen, Bergen, Norway; 136 Ophthalmology Department, Carol Davila University of Medicine and Pharmacy, Bucharest, Romania; 137 Ophthalmology Department, Emergency University Hospital Bucharest, Bucuresti, Romania; 138 2nd University Ophthalmology Department, Aristotle University of Thessaloniki, Thessaloniki, Greece; 139 Ophthalmology Department, University of Thessaly, Larissa, Greece; 140 Department of Community Medicine, Chettinad Hospital and Research Institute, Chettinad Academy of Research and Education, Chennai, India; 141 Health Research Section, Nepal Health Research Council, Kathmandu, Nepal; 142 Center of Complexity Sciences, National Autonomous University of Mexico, Mexico City, Mexico; 143 Faculty of Veterinary Medicine and Zootechnics, Autonomous University of Sinaloa, Culiacán Rosales, Mexico; 144 Department of Medicine, Can Tho University of Medicine and Pharmacy, Can Tho, Viet Nam; 145 Department of Medicine, Pham Ngoc Thach University of Medicine, Ho Chi Minh City, Viet Nam; 146 Department of Conservative Dentistry with Endodontics, Medical University of Silesia, Katowice, Poland; 147 Department of Ophthalmology and Visual Sciences, University of Michigan, Ann Arbor, MI USA; 148 Institute for Health Care Policy and Innovation, University of Michigan, Ann Arbor, MI USA; 149 Department of Epidemiology and Medical Statistics, University of Ibadan, Ibadan, Nigeria; 150 Faculty of Public Health, University of Ibadan, Ibadan, Nigeria; 151 Faculty of Medicine, University of Tripoli, Tripoli, Libya; 152 Ophthalmic Epidemiology Research Center, Shahroud University of Medical Sciences, Shahroud, Iran; 153 Department of Ophthalmology, University of California Los Angeles, Los Angeles, CA USA; 154 School of Medicine, Tehran University of Medical Sciences, Tehran, Iran; 155 Endocrinology and Metabolism Research Institute, Non-Communicable Diseases Research Center (NCDRC), Tehran, Iran; 156 Department of Nursing, Wollega University, Nekemte, Ethiopia; 157 Institute of Public Health, Charité Universitätsmedizin Berlin (Charité Medical University Berlin), Berlin, Germany; 158 Department of Ophthalmology, Isfahan University of Medical Sciences, Isfahan, Iran; 159 Emergency Department, Isfahan University of Medical Sciences, Isfahan, Iran; 160 Child Survival Unit, Centre for African Newborn Health and Nutrition, Ibadan, Nigeria; 161 Division of Ophthalmology, University of São Paulo, Ribeirão Preto, Brazil; 162 Department of Community Medicine, All India Institute of Medical Sciences, Nagpur, India; 163 Department of Midwifery, Adigrat University, Adigrat, Ethiopia; 164 Postgraduate Program in Epidemiology, Federal University of Rio Grande do Sul, Porto Alegre, Brazil; 165 Department of Clinical Pharmacy, Haramaya University, Harar, Ethiopia; 166 Toxicology Department, Shriram Institute for Industrial Research, Delhi, India; 167 Faculty of Medicine Health and Human Sciences, Macquarie University, Sydney, NSW Australia; 168 School of Medicine, Deakin University, Geelong, VIC Australia; 169 Independent Consultant, Santa Clara, CA USA; 170 Department of Pediatrics, Yonsei University College of Medicine, Seoul, South Korea; 171 Research Department, Electronic Medical Records for the Developing World, York, UK; 172 School of Biotechnology, Tan Tao University, Long An, Viet Nam; 173 Department of Health Promotion and Education, University of Ibadan, Ibadan, Nigeria; 174 Institute of Health Research, University of Health and Allied Sciences, Ho, Ghana; 175 Centre of Studies and Research, Ministry of Health, Muscat, Oman; 176 Department of Community Medicine, Manipal Academy of Higher Education, Mangalore, India; 177 Department of Economics, National Open University, Benin City, Nigeria; 178 Les Ophtalmologistes Associés de Monastir (The Associated Ophthalmologists of Monastir), Monastir, Tunisia; 179 Save Sight Institute, University of Sydney, Sydney, NSW Australia; 180 Sydney Eye Hospital, South Eastern Sydney Local Health District, Sydney, NSW Australia; 181 School of Health Professions and Human Services, Hofstra University, Hempstead, NY USA; 182 Department of Anesthesiology, Montefiore Medical Center, Bronx, NY USA; 183 Eye Research Center, Iran University of Medical Sciences, Tehran, Iran; 184 Health Policy Research Center, Shiraz University of Medical Sciences, Shiraz, Iran; 185 Department of Ophthalmology and Visual Sciences, University of Alabama at Birmingham, Birmingham, AL USA; 186 Research Department, Better Vision Foundation Nepal, Kathmandu, Nepal; 187 Global Consortium for Public Health Research, Datta Meghe Institute of Higher Education and Research, Wardha, India; 188 Department of Anthropology, Panjab University, Chandigarh, India; 189 Department of Health Policy and Strategy, Foundation for People-centric Health Systems, New Delhi, India; 190 SD Gupta School of Public Health, Indian Institute of Health Management Research University, Jaipur, India; 191 College of Optometry, Nova Southeastern University, Fort Lauderdale, FL USA; 192 Department of Public Health, Management and Science University, Shah Alam, Malaysia; 193 Jeffrey Cheah School of Medicine and Health Sciences, Monash University, Subang Jaya, Malaysia; 194 Department GF Ingrassia, University of Catania, Catania, Italy; 195 Renmin Hospital, Wuhan University, Wuhan, China; 196 University Centre Varazdin, University North, Varazdin, Croatia; 197 Department of Biomedical Sciences, Mercer University School of Medicine, Macon, GA USA; 198 Molecular Biology Unit, Sirius Training and Research Centre, Khartoum, Sudan; 199 Bio-Statistical and Molecular Biology Department, Sirius Training and Research Centre, Khartoum, Sudan; 200 Department of Pharmacology, Abadan School of Medical Sciences, Abadan, Iran; 201 Department of Optometry and Vision Sciences, Zahedan University of Medical Sciences, Zahedan, Iran; 202 Eye Research Center, Mashhad University of Medical Sciences, Mashhad, Iran; 203 Non-communicable Diseases Research Center, Tehran University of Medical Sciences, Tehran, Iran; 204 School of Medicine, Shahid Beheshti University of Medical Sciences, Tehran, Iran; 205 Department of Medicine, Democritus University of Thrace, Alexandroupolis, Greece; 206 College of Medicine and Public Health, Flinders University, Adelaide, Australia; 207 Department of Engineering, Western Sydney University, Sydney, NSW Australia; 208 Department of Dental Public Health, King Abdulaziz University, Jeddah, Saudi Arabia; 209 Department of Health Policy and Oral Epidemiology, Harvard University, Boston, MA USA; 210 Amity Institute of Forensic Sciences, Amity University, Noida, India; 211 Independent Consultant, Tehran, Iran; 212 Department of Internal Medicine, Ardabil University of Medical Science, Ardabil, Iran; 213 Department of Medical Laboratory Sciences, Adigrat University, Adigrat, Ethiopia; 214 Division of Cardiology, Massachusetts General Hospital, Boston, MA USA; 215 Department of Medical Engineering, University of South Florida, Tampa, FL USA; 216 Department of Applied Economics and Quantitative Analysis, University of Bucharest, Bucharest, Romania; 217 Department of Psychiatry and Behavioural Neurosciences, McMaster University, Hamilton, ON Canada; 218 Department of Psychiatry, University of Lagos, Lagos, Nigeria; 219 Department of Nursing Science, Bowen University, Iwo, Nigeria; 220 Department of Biology, Khalifa University, Abu Dhabi, United Arab Emirates; 221 School of Medicine, Western Sydney University, Campbelltown, NSW Australia; 222 Department of Optometry and Vision Science, University of KwaZulu-Natal, KwaZulu-Natal, South Africa; 223 Department of Forensic Medicine and Toxicology, Kasturba Medical College, Mangalore, Mangalore, India; 224 Privatpraxis, Heidelberg, Germany; 225 Yonsei University College of Medicine, Seodaemun-gu, South Korea; 226 Global Health Governance Programme, University of Edinburgh, Edinburgh, UK; 227 School of Dentistry, University of Leeds, Leeds, UK; 228 Department of Development Studies, International Institute for Population Sciences, Mumbai, India; 229 Medical School, Pham Ngoc Thach University of Medicine, Ho Chi Minh City, Viet Nam; 230 Department of Maternal and Child Nursing and Public Health, Federal University of Minas Gerais, Belo Horizonte, Brazil; 231 Department of Neonatology, Case Western Reserve University, Cleveland, OH USA; 232 Department of Health Sciences, Cihan University Sulaimaniya, Sulaymaniyah, Iraq; 233 Cihan University Sulaimaniya Research Center (CUSRC), Sulaymaniyah, Iraq; 234 Department of Population Science and Human Resource Development, University of Rajshahi, Rajshahi, Bangladesh; 235 College of Pharmaceutical Sciences, Andhra University, Visakhapatnam, India; 236 Department of Primary Care and Public Health, Imperial College London, London, UK; 237 Academic Public Health England, Public Health England, London, UK; 238 Department of Labour, Directorate of Factories, Government of West Bengal, Kolkata, India; 239 Sharjah Institute for Medical Research, University of Sharjah, Sharjah, United Arab Emirates; 240 Multidisciplinary Laboratory School of Health Sciences (FUSH), Foundation University, Islamabad, Pakistan; 241 International Center of Medical Sciences Research (ICMSR), Islamabad, Pakistan; 242 Faculty of Medicine, Bioscience and Nursing, MAHSA University, Selangor, Malaysia; 243 Interdisciplinary Research Centre in Biomedical Materials (IRCBM), COMSATS Institute of Information Technology, Lahore, Pakistan; 244 Ophthalmic Epidemiology Research Center, Shahid Beheshti University of Medical Sciences, Tehran, Iran; 245 Ophthalmic Research Center (ORC), Shahid Beheshti University of Medical Sciences, Tehran, Iran; 246 Ludwig Maximilian University of Munich, Munich, Germany; 247 Institute for Employment Research, Nuremberg, Germany; 248 College of Medicine, University of Sharjah, Sharjah, United Arab Emirates; 249 Faculty of Pharmacy, Mansoura University, Mansoura, Egypt; 250 Department of Anatomy, Ras Al Khaimah Medical and Health Sciences University, Ras Al Khaimah, United Arab Emirates; 251 Department of Entomology, Ain Shams University, Cairo, Egypt; 252 Medical Ain Shams Research Institute (MASRI), Ain Shams University, Cairo, Egypt; 253 Department of Pharmacology and Research, All India Institute of Medical Sciences, Jodhpur, India; 254 Indira Gandhi Medical College and Research Institute, Puducherry, India; 255 National Heart, Lung, and Blood Institute, National Institute of Health, Rockville, MD USA; 256 Independent Consultant, Karachi, Pakistan; 257 Amity Institute of Public Health, Amity University, Noida, India; 258 Department of Nursing, Arba Minch University, Arba Minch, Ethiopia; 259 Department of Ophthalmology, Harvard Medical School, Boston, MA USA; 260 Department of Veterinary Public Health and Preventive Medicine, Usmanu Danfodiyo University, Sokoto, Nigeria; 261 Unit of Basic Medical Sciences, University of Khartoum, Khartoum, Sudan; 262 Department of Medical Microbiology and Infectious Diseases, Erasmus University, Rotterdam, Netherlands; 263 School of Medicine, University of Alabama at Birmingham, Birmingham, AL USA; 264 Medicine Service, US Department of Veterans Affairs (VA), Birmingham, AL USA; 265 Department of Nursing, Dire Dawa University, Dire Dawa, Ethiopia; 266 Directive Board, Associação de Profissionais Licenciados de Optometria (Association of Licensed Optometry Professionals), Linda-a-Velha, Portugal; 267 Department of Medical Informatics, Mashhad University of Medical Sciences, Mashhad, Iran; 268 Clinial Research Development Unit, Mashhad University of Medical Sciences, Mashhad, Iran; 269 Department of Pharmacology, Shaqra University, Shaqra, Saudi Arabia; 270 Faculty of Public Health, Universitas Sam Ratulangi, Manado, Indonesia; 271 Department of Public Health, Arba Minch College of Health Sciences, Arba Minch, Ethiopia; 272 Department of Medicine, University of Crete, Heraklion, Greece; 273 Department of Nursing, Aksum University, Aksum, Ethiopia; 274 Urmia University of Medical Sciences, Urmia, Iran; 275 Division of Cardiology, Johns Hopkins University, Baltimore, MD USA; 276 Department of Epidemiology and Biostatistics, Bahir Dar University, Bahir Dar, Ethiopia; 277 Department of Community Medicine, Rajarata University of Sri Lanka, Anuradhapura, Sri Lanka; 278 Department of Pediatrics, Kyung Hee University, Seoul, South Korea; 279 Macquarie Medical School, Macquarie University, Sydney, NSW Australia; 280 Department of Mathematics, Imperial College London, London, UK

**Keywords:** Epidemiology, Optic nerve diseases

## Abstract

**Objectives:**

To estimate global and regional trends from 2000 to 2020 of the number of persons visually impaired by glaucoma and their proportion of the total number of vision-impaired individuals.

**Methods:**

A systematic review and meta-analysis of published population studies and grey literature from 2000 to 2020 was carried out to estimate global and regional trends in number of people with vision loss due to glaucoma. Moderate or severe vision loss (MSVI) was defined as visual acuity of 6/60 or better but <6/18 (moderate) and visual acuity of 3/60 or better but <6/60 (severe vision loss). Blindness was defined as presenting visual acuity <3/60.

**Results:**

Globally, in 2020, 3.61 million people were blind and nearly 4.14 million were visually impaired by glaucoma. Glaucoma accounted for 8.39% (95% uncertainty intervals [UIs]: 6.54, 10.29) of all blindness and 1.41% (95% UI: 1.10, 1.75) of all MSVI. Regionally, the highest proportion of blindness relating to glaucoma was found in high-income countries (26.12% [95% UI: 20.72, 32.09]), while the region with the highest age-standardized prevalence of glaucoma-related blindness and MSVI was Sub-Saharan Africa. Between 2000 and 2020, global age-standardized prevalence of glaucoma-related blindness among adults ≥50 years decreased by 26.06% among males (95% UI: 25.87, 26.24), and by 21.75% among females (95% UI: 21.54, 21.96), while MSVI due to glaucoma increased by 3.7% among males (95% UI: 3.42, 3.98), and by 7.3% in females (95% UI: 7.01, 7.59).

**Conclusions:**

Within the last two decades, glaucoma has remained a major cause of blindness globally and regionally.

## Introduction

Glaucoma refers to a group of ocular conditions united by a clinically characteristic optic neuropathy [1]. Although treatment by lowering intraocular pressure (IOP) can arrest or slow its deterioration in most cases, glaucoma remains the most common cause of irreversible blindness worldwide [2]. The more common chronic forms of glaucoma are usually asymptomatic until later stages of the disease, when individuals may present with advanced visual field loss and can progress to complete loss of vision. Primary open-angle glaucoma (POAG), is the most common subtype and risk factors include age, elevated IOP, sub-Saharan African ethnic origin, positive family history, and high myopia. Primary angle-closure glaucoma (PACG), is a visually devastating form characterised by an ocular anatomical predisposition particularly prevalent in East Asians.

A meta-analysis by Tham et al. in 2013, using data from 50 population-based studies that used case definitions based on specific structural or functional evidence of glaucomatous optic neuropathy, estimated the number of people (aged 40-80 years) with glaucoma worldwide to be 64.3 million, increasing to 76.0 million in 2020 and 111.8 million in 2040 [3]. A more recent meta-analysis estimated that POAG affected 68.56 million (95% confidence interval (95% CI), 59.99–79.98), 2.4% (95% CI, 2.0–2.8) of the global population older than 40 years [4]. However, for individuals and society, the burden of a disease is more important than just the presence of a disease including its early subjectively asymptomatic stages. Often population-based glaucoma studies did not report on the number of people blind or visually impaired due to glaucoma; however, over the last few decades, many more population-based eye surveys have been conducted with improved methods of glaucoma case detection and reporting of glaucoma-related vision loss. Using data from population-based studies conducted between 1980-2012 and collated within the Global Vision Database, the Vision Loss Expert Group (VLEG; an international ophthalmic epidemiology reference group) working with the Global Burden of Disease Study reported on the number of individuals visually impaired or blind due to glaucoma, examined regional differences and for the first time reported the temporal changes for the period from 1990 to 2012. Using an ongoing systematic literature review, VLEG has continued to update the Global Vision Database with more population-based datasets. In this current meta-analysis, we (VLEG) report the number of people affected by glaucoma-related blindness and MSVI in 2020, the change between 2000 and 2020, and differences by region and sex.

## Methods

The first stage of data preparation included a systematic review of published (between Jan 1, 1980, and Oct 1, 2018) population-based studies of vision impairment and blindness by the VLEG that also included grey literature sources. Eligible studies from this review were combined with data from Rapid Assessment of Avoidable Blindness (RAAB) studies. Data from the US National Health and Nutrition Examination Survey and the World Health Organization (WHO) Study on Global Ageing and Adult Health were provided by the GBD team. More detailed methods are published elsewhere [5, 6] and briefly discussed as follows.

In total, the VLEG identified 137 studies and extracted data from 70 studies in their 2010 review, and an additional 67 studies in their 2014–18 review. Studies were primarily national and subnational cross-sectional surveys. Additionally, the VLEG commissioned the preparation of 5-year age-disaggregated RAAB data from the RAAB repository [7]. Studies were included if they met the following criteria: visual acuity measured using a test chart that could be mapped to the Snellen scale, and a sample that was representative of the population. Self-report of vision loss was excluded. We used the International Classification of Diseases 11th edition criteria for vision loss, as applied by the WHO, which categorizes people according to vision in the better eye on presentation [8]. Moderate vision loss is defined as a visual acuity of 6/60 or better but less than 6/18, severe vision loss as a visual acuity of 3/60 or better but less than 6/60, and blindness as a visual acuity of less than 3/60 or less than 10° visual field around central fixation; however, in practice, visual field data from population-based eye surveys are scarce.

First, we separated raw data into vision-loss envelopes for all-cause moderate, and severe vision loss, and blindness. Data were input into a mixed-effects meta-regression tool developed by the Institute for Health Metrics and Evaluation (IHME) called MR-BRT (meta-regression; Bayesian; regularised; trimmed) [9]. Presenting vision impairment was the reference definition for each level of severity. Undercorrected refractive error data were extracted directly from data sources where available and otherwise calculated by subtracting best-corrected vision impairment from presenting vision impairment prevalence for each level of severity in studies that reported both measures for a given location, sex, age group, and year. All other causes were quantified as part of the best-corrected estimates of vision impairment at each level of severity.

We modelled distance vision impairment and blindness due to the following causes: cataracts, undercorrected refractive error, age-related macular degeneration, myopic macular degeneration, glaucoma, diabetic retinopathy, and other causes of vision impairment (in aggregate). The minimum age for inclusion of data for these causes was set at 20 years for cataract and diabetic retinopathy, and 45 years for glaucoma and age-related macular degeneration. Other vision impairment estimates were combined with less prevalent causes of vision impairment to create a residual category (e.g., retinopathy of prematurity, vitamin A deficiency, trachoma).

We produced location, year, age, and sex-specific estimates of MSVI and blindness using Disease Modelling Meta-Regression (Dismod-MR) 2.1 [10]. The data processing steps are described elsewhere [6]. Briefly, Dismod-MR 2.1 models were run for all vision impairment by severity (moderate, severe, blindness) regardless of cause and, separately, for MSVI and blindness due to each modelled cause of vision impairment (e.g., MSVI due to cataract and blindness due to cataract). Then, models of MSVI due to specific causes were split into moderate and severe estimates using the ratio of overall prevalence in the all-cause moderate presenting vision impairment and severe presenting vision impairment models. Next, prevalence estimates for all causes by severity were scaled to the models of all-cause prevalence by severity. This produced final estimates by age, sex, year, and location for each individual cause of vision impairment by severity. We age-standardized our estimates using the GBD standard population [11].

## Results

According to our estimates from 2020, approximately 3.61 million (95% uncertainty intervals (UIs): 2.81, 4.42) people were blind and 4.14 million (95% UI: 3.24, 5.18) were visually impaired (MSVI) globally because of glaucoma (Table 1). An estimated 1.89 million males and 1.72 million females of all ages, and 1.89 million males and 1.71 million females aged ≥50 years were blind due to glaucoma in 2020 (Table 2). The number of males and females (all ages) with glaucoma-related MSVI in 2020 was 2.00 million and 2.14 million, respectively, whereas an estimated 2.00 million and 2.14 million people were aged 50 years and over (Table 3).

**Table 1:** Number of people (mean [95% UI]) with blindness (presenting visual acuity <3/60) or MSVI (presenting visual acuity <6/18, ≥3/60) due to glaucoma, the age-standardized prevalence (%) in people aged ≥50 years (mean [95% UI]), and the percentage of all blindness or MSVI attributed to glaucoma (95% UI) globally and in GBD super-regions in 2020.

		Blindness due to glaucoma in 2020			MSVI due to glaucoma in 2020		
Global/GBD Super-region	Total population in 2020 ('000s)	Number of people (‘000 s) *(all ages)*	Age-standardized prevalence (%) ( ≥ *50 years*)	Percentage contribution by glaucoma to all cases of blindness ( ≥ *50 years*)	Number of people (‘000 s) (*all ages*)	Age-standardized prevalence (%) ( ≥ *50 years*)	Percentage contribution by glaucoma to all MSVI cases ( ≥ *50 years*)
Global	7,890,000	3,608 (2812, 4423)	0.20 (0.16–0.25)	8.39 (6.54–10.29)	4145 (3243, 5176)	0.23 (0.18–0.29)	1.41 (1.10–1.75)
Southeast Asia, East Asia, and Oceania	2,192,710	757 (577, 961)	0.13 (0.10–0.17)	5.02 (3.83–6.37)	1163 (917, 1452)	0.20 (0.16–0.25)	1.40 (1.10–1.75)
Central Europe, Eastern Europe, and Central Asia	417,291	178 (139, 220)	0.12 (0.10–0.15)	12.59 (9.80–15.49)	213 (167, 270)	0.15 (0.12–0.19)	1.19 (0.93–1.50)
High-income	1,087,856	786 (623, 965)	0.14 (0.11–0.17)	26.12 (20.72–32.09)	596 (468, 763)	0.11 (0.09–0.14)	1.92 (1.51–2.46)
Latin America and Caribbean	601,551	335 (257, 412)	0.26 (0.20–0.32)	9.15 (7.01–11.25)	499 (391, 623)	0.39 (0.30–0.48)	2.04 (1.60–2.55)
North Africa and Middle East	631,727	463 (355, 579)	0.57 (0.44–0.71)	14.98 (11.47–18.72)	326 (252, 420)	0.38 (0.29–0.48)	1.49 (1.15–1.92)
South Asia	1,841,435	579 (441, 729)	0.23 (0.17–0.28)	4.85 (3.69–6.10)	955 (747, 1200)	0.34 (0.27–0.42)	0.99 (0.78–1.25)
Sub-Saharan Africa	1,114,806	511 (400, 629)	0.66 (0.52–0.81)	10.05 (7.86–12.37)	392 (308, 494)	0.46 (0.36–0.57)	1.92 (1.51–2.42)

**Table 2:** Number of males and females with glaucoma blindness (presenting visual acuity <3/60 attributed to glaucoma), and age-standardized prevalence (% [95% UI]) for all ages and for people aged ≥50 years in 2020.

		Total number with glaucoma blindness and age-standardized glaucoma blindness in 2020 (all ages)				Total number with glaucoma blindness and age-standardized glaucoma blindness in people aged 50+ years in 2020			
Global/GBD Super-region	Total population in 2020 ('000s)	Number of males (000 s)	Number of females (000s)	Age-standardized prevalence (%) in males	Age-standardized prevalence (%) in females	Number of males (000 s)	Number of females (000 s)	Age-standardized prevalence (%) in males	Age-standardized prevalence (%) in females
Global	7,890,000	1892 (1491, 2315)	1717 (1324, 2110)	0.05 (0.04–0.07)	0.04 (0.03-0.05)	1887 (1487, 2310)	1713 (1322, 2107)	0.24 (0.19–0.30)	0.17 (0.13–0.21)
Southeast Asia, East Asia, and Oceania	2,192,710	387 (296, 491)	370 (281, 470)	0.03 (0.03–0.04)	0.03 (0.02-0.03)	385 (294, 489)	369 (280, 469)	0.15 (0.11–0.19)	0.12 (0.09–0.15)
Central Europe, Eastern Europe, and Central Asia	417,291	79 (62, 96)	100 (77, 123)	0.03 (0.03–0.04)	0.02 (0.02-0.03)	78 (61, 96)	100 (77, 123)	0.16 (0.13–0.19)	0.11 (0.08–0.13)
High-income	1,087,856	376 (301, 456)	410 (318, 511)	0.04 (0.03–0.04)	0.03 (0.02-0.03)	376 (301, 456)	409 (317, 510)	0.17 (0.13–0.20)	0.12 (0.10–0.15)
Latin America and Caribbean	601,551	172 (132, 211)	163 (124, 201)	0.07 (0.05–0.08)	0.05 (0.04-0.06)	171 (132, 211)	163 (123, 201)	0.31 (0.24–0.38)	0.23 (0.17–0.28)
North Africa and Middle East	631,727	267 (205, 333)	196 (148, 248)	0.15 (0.12–0.19)	0.10 (0.08-0.13)	266 (205, 332)	196 (148, 248)	0.69 (0.53–0.85)	0.46 (0.35–0.58)
South Asia	1,841,435	339 (260, 427)	240 (181, 303)	0.06 (0.05–0.08)	0.04 (0.03-0.05)	338 (259, 425)	239 (181, 302)	0.28 (0.21–0.35)	0.18 (0.13–0.22)
Sub-Saharan Africa	1,114,806	273 (215, 335)	238 (185, 295)	0.17 (0.14–0.21)	0.12 (0.10-0.15)	272 (215, 335)	238 (185, 294)	0.79 (0.62–0.96)	0.56 (0.44–0.69)

**Table 3:** Number of males and females with glaucoma MSVI, and the age-standardized prevalence (% [95% uncertainty intervals (UIs)]) of glaucoma MSVI (all ages and people aged ≥ 50 years) in 2020.

	Total population	Total number with glaucoma MSVI and age-standardized glaucoma MSVI in 2020 (all ages)				Total number with glaucoma MSVI and age-standardized glaucoma MSVI in people aged 50+ years in 2020			
Global/GBD Super-region	2020, total population (000 s)	Number of males with glaucoma MSVI in 2020 (000 s)	Number of females with glaucoma MSVI in 2020 (000 s)	Age standardized prevalence of MSVI in males in 2020	Age standardized prevalence of MSVI in females in 2020	Number of males (50+ years) with glaucoma MSVI in 2020 (000 s)	Number of females (50+ years) with glaucoma MSVI in 2020 (000 s)	Age standardized prevalence of MSVI in males in 2020	Age standardized prevalence of MSVI in females in 2020
Global	7,890,000	2004 (1575, 2511)	2140 (1679, 2677)	0.05 (0.04–0.07)	0.05 (0.04–0.06)	1999 (1571, 2508)	2136 (1676, 2671)	0.25 (0.19–0.31)	0.21 (0.17–0.27)
Southeast Asia, East Asia, and Oceania	2,192,710	555 (438, 693)	608 (477, 768)	0.05 (0.04–0.06)	0.04 (0.03–0.05)	554 (437, 691)	606 (476, 766)	0.21 (0.16–0.26)	0.19 (0.15–0.25)
Central Europe, Eastern Europe, and Central Asia	417,291	78 (60, 99)	135 (106, 172)	0.03 (0.03–0.04)	0.03 (0.03–0.04)	78 (60, 99)	135 (106, 172)	0.15 (0.12–0.19)	0.15 (0.11–0.18)
High-income	1,087,856	249 (195, 319)	347 (272, 447)	0.02 (0.02–0.03)	0.02 (0.02–0.03)	249 (194, 318)	347 (272, 447)	0.11 (0.09–0.14)	0.11 (0.08–0.14)
Latin America and Caribbean	601,551	246 (194, 308)	252 (197, 317)	0.09 (0.07–0.12)	0.08 (0.06–0.10)	246 (193, 307)	252 (197, 317)	0.43 (0.34–0.53)	0.35 (0.28–0.44)
North Africa and Middle East	631,727	169 (130, 217)	158 (121, 203)	0.09 (0.07–0.11)	0.08 (0.06–0.10)	168 (130, 216)	157 (120, 202)	0.40 (0.31–0.52)	0.35 (0.27–0.45)
South Asia	1,841,435	515 (401, 649)	440 (343, 554)	0.08 (0.07–0.10)	0.07 (0.05–0.08)	514 (400, 648)	439 (342, 552)	0.38 (0.30–0.47)	0.30 (0.24–0.38)
Sub-Saharan Africa	1,114,806	192 (151, 243)	200 (157, 251)	0.11 (0.09–0.13)	0.09 (0.07–0.12)	192 (150, 242)	199 (157, 250)	0.49 (0.39–0.61)	0.43 (0.34–0.53)

Glaucoma caused 8.39% (95% UI: 6.54, 10.29) of all blindness in 2020 worldwide. Regionally, the highest proportion of blindness attributed to glaucoma was found in high-income countries (26.12% [95% UI: 20.72, 32.09]) and North Africa and Middle East (14.98% [95% UI: 11.47, 18.72]) (Table 1). The regions with the lowest proportion of glaucoma-related blindness of all blind individuals were South Asia (4.85% [UI: 3.69, 6.10]), and Southeast Asia, East Asia, and Oceania (5.02% [95% UI: 3.83, 6.37]). Glaucoma caused 1.41% (95% UI: 1.10, 1.75) of all cases with MSVI in 2020 worldwide. Latin America and Caribbean (2.04% [95% UI: 1.60, 2.55]), high-income countries (1.92% [95% UI: 1.51, 2.46]) and Sub-Saharan Africa (1.92% [95% UI: 1.51, 2.42]) were regions with the highest percentage of glaucoma-related MSVI of all individuals with MSVI (Table 1).

In 2020, the global age-standardized prevalence of glaucoma-related blindness in those aged ≥50 years was 0.20% (95% UI: 0.16, 0.25) and for glaucoma-related MSVI was 0.23% (95% UI: 0.18, 0.29) (Table 1). The region with the highest age-standardized prevalence of glaucoma-related blindness was Sub-Saharan Africa (0.66% [95% UI: 0.52, 0.81]). The lowest age-standardized prevalence of glaucoma blindness in 2020 was in the regions of Central Europe, Eastern Europe, and Central Asia (0.12% (95% UI: 0.10, 0.15). The regions with the highest age-standardized prevalence of glaucoma-related MSVI in 2020 were Sub-Saharan Africa (0.46% [95% UI: 0.36, 0.57]), and Latin America and the Caribbean (0.39% [95% UI: 0.30, 0.48]). The lowest figures were found in high-income countries (0.11% [95% UI: 0.09, 0.14]) and Central Europe, Eastern Europe, and Central Asia (0.15% [95% UI: 0.12, 0.19]) (Table 1).

Between 2000 and 2020, the global percentage change in age-standardized prevalence of glaucoma-related blindness among adults ≥50 years improved by 26.06% (95% UI: 25.87, 26.24) among males and by 21.75% in females (95% UI: 21.54, 21.96) (Table 4). A large reduction (26-39%) in the age-standardized prevalence of glaucoma-related blindness amongst adults aged ≥50 years (both sexes) was found in Southeast Asia, East Asia and Oceania (−38.99% [95% UI: −39.16, −38.82]), South Asia (−35.75% [95% UI: −35.93, −35.57]), North Africa and Middle East (−30.04% [95% UI: −30.23, −29.84]), and Central Europe, Eastern Europe, and Central Asia (−26.62% [95% UI: −26.81, −26.43]) with more modest reductions in Latin America and the Caribbean (−14.47% [95% UI: −14.71, −14.24]), Sub-Saharan Africa (−16.85% [95% UI: −17.06, −16.64]) and high-income countries (-8.71% [95% UI: -8.95, -8.48]) (Table 4). There were minimal differences between males and females in respect of the reduction in age-standardized prevalence of glaucoma blindness across regions (Table 4).

**Table 4:** Change in the burden of glaucoma blindness (presenting visual acuity <3/60, attributed to glaucoma) in adults aged 50 years and older between 2000 and 2020.

Global/GBD Super-region	Crude Prevalence			Percentage change in number of cases			Percentage change in age standardized prevalence		
	Male (%, 95% UI)	Female (%, 95% UI)	Both (%, 95% UI)	Male (%, 95% UI)	Female ((%, 95% UI)	Both (%, 95% UI)	Male (%, 95% UI)	Female (%, 95% UI)	Both (%, 95% UI)
**Global**	−20.30 (−20.50–−20.09)	−19.42 (−19.64–−19.21)	−19.87 (−20.08–−19.66)	40.57 (40.20–40.93)	41.55 (41.17–41.93)	41.03 (40.66–41.41)	−26.06 (−26.24–−25.87)	−21.75 (−21.96–−21.54)	−23.34 (−23.54–−23.14)
**High income countries**	10.01 (9.74–−10.28)	1.55 (1.27–1.83)	5.38 (5.11–5.65)	61.89 (61.49–62.28)	41.45 (41.06–41.84)	50.55 (50.16–50.94)	−9.88 (−10.10–−9.66)	−9.86 (−10.10–−9.61)	−8.71 (−8.95–−8.48)
**Central Europe, Eastern Europe and Central Asia**	−16.23 (−16.46–−16.01)	−21.10 (−21.32–−20.89)	−19.01 (−19.24–−18.79)	8.66 (8.36–8.95)	−2.39 (−2.66–−2.12)	2.18 (1.90–2.46)	−25.19 (−25.38–−25.00)	−28.64 (−28.83–−28.45)	−26.62 (−26.81–−26.43)
**Latin America and Caribbean**	−10.22 (−10.46–−9.97)	−2.97 (−3.24–−2.69)	−7.06 (−7.32–−6.81)	76.93 (76.44–77.41)	98.61 (98.05–99.16)	86.87 (86.36–87.39)	−16.17 (−16.40–−15.94)	−11.90 (−12.15–−11.65)	−14.47 (−14.71–−14.24)
**North Africa and Middle East**	−34.60 (−34.79–−34.42)	−27.97 (−28.19–−27.76)	−31.96 (−32.15–−31.76)	35.88 (35.50–36.26)	49.88 (49.43–50.33)	41.48 (41.08–41.89)	−29.42 (−29.61–−29.22)	−29.33 (−29.54–−29.12)	−30.04 (−30.23–−29.84)
**South Asia**	−27.90 (−28.11–−27.69)	−29.29 (−29.50–−29.08)	36.07 (35.67–36.47)	41.02 (40.59–41.45)	38.08 (37.67–38.49)	−32.90 (−33.08–−32.71)	−37.45 (−37.63–−37.27)	−35.75 (−35.93–−35.57)	
**Southeast Asia, East Asia and Oceania**	−35.28 (−35.47–−35.09)	−35.62 (−35.81–−35.43)	−35.49 (−35.68–−35.30)	27.97 (27.60–28.35)	31.34 (30.95–31.72)	29.60 (29.22–29.98)	−41.07 (−41.24–−40.91)	−37.77 (−37.94–−37.59)	−38.99 (−39.16–−38.82)
**Sub-Saharan Africa**	−21.52 (−21.73–−21.32)	−21.38 (−21.59–−21.16)	−21.91 (−22.11–−21.70)	38.84 (38.47–39.20)	52.53 (52.12–52.94)	44.89 (44.51–45.28)	−17.82 (−18.03–−17.61)	−15.16 (−15.38–−14.93)	−16.85 (−17.06–−16.64)

Between 2000 and 2020, the global percentage change in age-standardized prevalence of glaucoma MSVI among adults (≥50 years) increased among males (3.7% [95% UI: 3.42, 3.98]) and females (7.3% [95% UI: 7.01, 7.59]) (Table 5). Southeast Asia, East Asia and Oceania were the only world regions where a substantial increase in the age-standardized prevalence of glaucoma MSVI was observed for both sexes (18.43%, [95% UI: 18.11, 18.75]) with more modest increases in Sub-Saharan Africa (1.71% [95% UI: 1.44, 1.98]) and high-income countries (1.65% [95% UI: 1.36, 1.95]). Reductions in glaucoma MSVI were noted in the other regions where the reduction was slightly greater among females than males. In Sub-Saharan Africa, the increase in the age-standardized prevalence of glaucoma-related MSVI was much greater among females (3.24% [95% UI:2.96, 3.52]) than among males (0.75% [95% UI:0.48, 1.02]).

**Table 5:** Change in the burden of glaucoma MSVI (presenting visual acuity <6/18 but ≥3/60 attributed to glaucoma) in adults aged 50 years and older between 2000 and 2020.

Global/GBD Super-region	Crude Prevalence			Percentage change in number of cases			Percentage change in age standardized prevalence		
	Male (%, 95% UI)	Female (%, 95% UI)	Both (%, 95% UI)	Male (%, 95% UI)	Female (%, 95% UI)	Both (%, 95% UI)	Male (%, 95% UI)	Female (%, 95% UI)	Both (%, 95% UI)
**Global**	9.57 (9.26–9.87)	8.55 (8.25–8.85)	9.04 (8.74–9.34)	93.24 (92.71–93.77)	90.69 (90.17–91.22)	91.92 (91.39–92.44)	3.70 (3.42–3.98)	7.30 (7.01–7.59)	5.94 (5.66–6.23)
**High income countries**	22.94 (22.58–23.30)	11.19 (10.86–11.53)	15.34 (15.00–15.68)	80.92 (80.38–81.45)	54.88 (54.42–55.35)	64.78 (64.29–65.27)	2.00 (1.70–2.29)	1.35 (1.05–1.64)	1.65 (1.36–1.95)
**Central Europe, Eastern Europe and Central Asia**	7.17 (6.85–7.49)	3.02 (2.71–3.32)	4.18 (3.87–4.48)	39.01 (38.59–39.42)	27.45 (27.08–27.83)	31.44 (31.06–31.83)	−1.72 (−2.00–−1.43)	−3.00 (−3.28–−2.72)	−2.65 (−2.93–−2.37)
**Latin America and Caribbean**	−0.37 (−0.64–−0.10)	−0.71 (−0.98–−0.43)	−0.65 (−0.92–−0.38)	96.34 (95.80–96.87)	103.24 (102.67–103.80)	99.77 (99.23–100.31)	−4.79 (−5.05–−4.53)	−6.32 (−6.57–−6.06)	−5.75 (−6.01–−5.50)
**North Africa and Middle East**	−16.22 (−16.46–−15.97)	−11.59 (−11.86–−11.33)	−14.05 (−14.30–−13.79)	74.08 (73.56–74.59)	83.97 (83.42–84.52)	78.72 (78.19–79.25)	−9.88 (−10.15–−9.62)	−10.12 (−10.38–−9.85)	−10.23 (−10.50–−9.96)
**South Asia**	−2.66 (−2.93–−2.38)	−6.01 (−6.29–−5.74)	−4.46 (−4.73–−4.18)	83.71 (83.18–84.24)	90.01 (89.46–90.57)	86.56 (86.03–87.10)	−7.48 (−7.73–−7.23)	−12.20 (−12.44–−11.96)	−10.15 (−10.39–−9.90)
**Southeast Asia, East Asia and Oceania**	23.41 (23.07–23.75)	21.88 (21.54–22.22)	22.65 (22.31–22.98)	144.01 (143.33–144.68)	148.63 (147.93–149.32)	146.40 (145.73–147.07)	15.89 (15.58–16.19)	19.94 (19.62–20.27)	18.43 (18.11–18.75)
**Sub-Saharan Africa**	−4.09 (−4.36–−3.82)	−2.79 (−3.06–−2.53)	−3.63 (−3.90–−3.36)	69.67 (69.20–70.14)	88.57 (88.06–89.09)	78.81 (78.31–79.30)	0.75 (0.48–1.02)	3.24 (2.96–3.52)	1.71 (1.44–1.98)

## Discussion

Glaucoma was the cause for blindness in 3.61 million people or 8.4% of the 43.3 million blind people globally in 2020, and glaucoma was the cause for MSVI in 4.14 million people or 1.4% of the 295 million people visually impaired in 2020 [6]. Our previous publication on vision loss due to glaucoma in 2010 reported lower numbers affected by blindness (2.1 million) and similar numbers with MSVI (4.2 million) [12]. In this most recent analysis, glaucoma was ranked as the second leading cause of blindness (after cataract) and fourth leading cause of MSVI, and therefore the most common cause of irreversible blindness, and the second most common cause of irreversible MSVI [6]. These figures highlight the importance of glaucoma as a public health concern, and it should be noted that these are almost certainly underestimations due to methodological issues where blindness prevalence surveys (in particular those of rapid design) often assign the most “treatable” disease as the primary cause of blindness assuming that cataract is more treatable than glaucoma. Furthermore, in advanced glaucoma, individuals may meet WHO perimetric-based definitions of blindness (<10 degrees of central field in the better eye) whilst retaining normal visual acuity. Hence, as visual field data is rarely collected in population-based surveys, a potentially large but unknown number of individuals are misclassified as visually unimpaired when they may be, in fact, glaucoma blind. Additionally, the definition of MSVI does not include perimetric criteria, though non-blinding visual field loss certainly results in vision impairment.

There is increasing global pressure on health-care resources due to the changing demographics of the population. The number of people aged 65 years and older is expected to increase from 700 million to 1.5 billion in the next 30 years, with the largest increases in low- and middle-income countries (LMICs). This will result in populations undergoing “epidemiological transition” and experiencing a higher prevalence of diseases associated with higher development and aging, including glaucoma [3, 13, 14]. Glaucoma disproportionately accounted for a greater proportion of blindness than it did for MSVI. The percentage of blindness caused by glaucoma showed regional variations, with relatively low figures in regions with relatively young populations such as South Asia, South-East Asia and Oceania, and Latin America and the Caribbean, and with relatively high figures in regions with relatively old populations such as the high-income regions (Table 1). Bucking this trend were the regions of North Africa and Middle East and Sub-Saharan Africa, both with younger populations than the median global age but with a relatively high contribution of glaucoma to blindness, most probably on account of these regions having the highest age-standardized prevalence of glaucoma-related blindness. Glaucoma causes a particular challenge as many LMICs experience these demographic changes. Due to the typically lengthy asymptomatic phase, it is well documented that in high-income countries, less than half of glaucoma is diagnosed [15, 16]. In LMICs, this proportion increases to over 90% and approximately 35% of patients are estimated to be blind at diagnosis [17–20]. Furthermore, the higher prevalences on the African continent are almost certainly due in part to lack of or insufficient access to treatment.

It is encouraging to note that between 2000 and 2020, the global percentage change in age-standardized prevalence of glaucoma-related blindness among adults ≥50 years has decreased (-26.06% [95% UI: -26.24, -25.87] among males and females (-21.75% [95% UI: -21.96, -21.54] (Table 4). We identified only two studies that have reported reductions in glaucoma-related blindness incidence, both from high-income countries. A recent analysis of the Finnish Register of Vision Impairment and a social insurance register found that the incidence of reported vision impairment in those with treated glaucoma had reduced by a third over 40 years, from 32/100,000 in the 1980s to 21/100,000 in the 2010-19 decade, with no sex differences [21]. This Finnish study reported that the proportion of overall vision impairment had increased in recent decades to approximately 50% suggesting better glaucoma care and earlier diagnosis, and this may be reflected in their finding that the age of onset of reported glaucoma-related vision impairment increased in more recent decades [21]. The Olmsted County (Minnesota, USA) population-based study reported that the 20-year probability of progression to glaucoma-related blindness in at least one eye had decreased from 26% for subjects diagnosed in 1965-1980 to 13% for those diagnosed in 1981-2000 [22]. In our analysis, reductions in age-standardized prevalence of glaucoma-related blindness between 2000-2020 in Latin America and Caribbean and Sub-Saharan Africa were not as marked as that for other LMIC regions. These data highlight the urgent need for improved eye health care in these regions, including a sustainable capacity to diagnose and treat glaucoma at earlier stages.

In all regions, in 2020, the age-standardized prevalence of glaucoma-related blindness was higher in males than in females. In Central Asia and high-income countries, the age-standardized prevalence of MSVI-related blindness was higher in females than in males, while in other regions there was no sex difference. Male gender has been found to be a significant risk factor for POAG in several studies [4, 23]. Women are well documented to be at a higher risk of PACG, the less prevalent but more blinding form of glaucoma [24]. The underlying reasons for the different gender-related predispositions are unclear but will be multifactorial, such as differential access to health systems.

Less encouraging than the temporal change in glaucoma blindness, was the finding that between 2000 and 2020, the mean global age-standardized prevalence of glaucoma-related MSVI among adults (≥50 years) had increased among males (3.70% [95% UI: 3.42, 3.98]) and females (7.30% [95% UI: 7.01, 7.59]) (Table 5). These increases took place mainly in Southeast Asia, East Asia and Oceania, Sub-Saharan Africa, and high-income countries. These data suggest a triaging phenomenon: although efforts to contain glaucoma blindness have had some success, the availability of current resources is not meeting the demands at visually significant but lower thresholds of visual impairment. The observed increase in glaucoma-related MSVI may be interpreted as an improvement if more cases are detected at an earlier stage before the onset of blindness.

The design of our study had potential limitations. First, as we discussed in our report of the global prevalence of vision loss, a significant limitation was that many countries lacked data across the time period, or that there was only sub-national data available [5]. Second, the majority of population-based studies within the database that reported on vision loss due to glaucoma did not disaggregate their reported findings into glaucoma diagnostic subtypes such as POAG and PACG; therefore, we could not differentiate between glaucoma subtypes in our analysis. Third, as mentioned above, protocol dictated that population-based studies will report one cause as the principal cause for an individual examined in that individual study so that causal prevalence can be calculated. In situations where multiple disorders contribute equally to visual loss, only the “most easily preventable” or the “most readily curable” cause is usually recorded [25] which underestimates the impact of diseases such as glaucoma and diabetic retinopathy. It may hold true in particular for patients with cataracts in which the ophthalmoscopical examination of the optic nerve is obscured leading to underdetection of glaucomatous optic nerve damage. Strengths of this study included a large amount of population-based data accessed and utilized and the trend analysis of causes of vision impairment and blindness, usage of non-linear age trends and modelling of data that were not reported by age, systematic quantitative analysis and reporting of uncertainty intervals. The large size of the network of ophthalmic researchers involved in first identification and then evaluation of data sources allowed access to unpublished materials and permitted us to obtain additional unpublished data from study investigators who had only published summary data, to evaluate all the major vision impairment studies, and to include only studies that met specific inclusion criteria regarding population representativeness and clear description and definition of visual acuity procedures.

Given the high prevalence of glaucoma and vision impairment secondary to glaucoma, the economic and social burden of the condition is substantial. Diagnosed individuals often require lifelong treatment and monitoring, which requires significant individual-level patient and carer support, for example, with adherence to treatment, and training of ophthalmologists and allied health professionals. Solutions to these challenges of diagnosis and ongoing monitoring may arrive through the innovative use of new community care models, availability of home monitoring of both intraocular pressure (IOP) and visual field function, and implementation of screening programs [26–28]. Early detection, both opportunistic and with targeted screening of those with higher genetic risk, is also important as is improved awareness of glaucoma. In addition to government systems, permissive regulations in some countries may be required to allow various models of health care delivery to function as well as possible. The recently published ‘Package of Eye Care Interventions’ by the World Health Organization provides a useful set of recommended, evidenced-based glaucoma care interventions with material resources required for implementation, health promotion and prevention, screening, diagnosis and monitoring, treatment, and rehabilitation [29]. These and other resources are necessary to facilitate policy-makers and technical decision-makers across countries to integrate glaucoma care into the packages and policies of their national health services.

## Summary

### What was known before

- Globally, in 2020, 3.61 million people were blind and nearly 4.14 million were visually impaired by glaucoma.

### What this study adds

- The contribution of glaucoma to blindness and moderate and severe vision impairment (MSVI) by region and the change in this contribution between 2000-2020 The change in global age-standardized prevalence of glaucoma-related blindness and MSVI between 2000 and 2020 and the differences by sex and region.

### Supplementary information

Appendix: Contributions by Authors

## Data Availability

Data sources for the Global Vision Database are listed at the following weblink http://www.anglia.ac.uk/verigbd. Fully disaggregated data is not available publicly due to data sharing agreements with some principal investigators yet requests for summary data can be made to the corresponding author.
