# Supplementary material for: Global estimates on the number of people blind or visually impaired by glaucoma: A meta-analysis from 2000 to 2020
Source: Eye (Lond). 2024 Apr 2;38(11):2036–46. doi: 10.1038/s41433-024-02995-5 (PMC11269708; doi:10.1038/s41433-024-02995-5)
Supplement: Supplementary file 1 — Appendix: Contributions by Authors [file 41433_2024_2995_MOESM1_ESM.docx]

**Appendix: Contributions by Authors**

# GBD 2019 Blindness and Vision Impairment Collaborators

### Providing data or critical feedback on data sources

Yohannes Habtegiorgis Abate, Mozhan Abdollahi, Richard Gyan Aboagye, Qorinah Estiningtyas Sakilah Adnani, Bright Opoku Ahinkorah, Danish Ahmad, Ayman Ahmed, Louay Almidani, Hubert Amu, Sofia Androudi, Jalal Arabloo, Tahira Ashraf, Sara Bagherieh, Ovidiu Constantin Baltatu, Mehmet Firat Baran, Amadou Barrow, Nebiyou Simegnew Bayileyegn, Jasvinder Singh Bhatti, Mukharram Bikbov, Rupert Bourne, Marija M Bozic, Tasanee Braithwaite, Paul Svitil Briant, Robert Casson, Muthia Cenderadewi, Vijay Kumar Chattu, Maria Vittoria Cicinelli, Xiaochen Dai, Ana Maria Dascalu, Meghnath Dhimal, Thao Huynh Phuong Do, Thanh Chi Do, Joshua R Ehrlich, Michael Ekholuenetale, Mohammad Hassan Emamian, Hossein Farrokhpour, Arthur G Fernandes, David S Friedman, João M Furtado, Bárbara Niegia Garcia Goulart, Mesay Dechasa Gudeta, Sapna Gupta, Vivek Kumar Gupta, Veer Bala Gupta, Sung Hwi Hong, Hong-Han Huynh, Segun Emmanuel Ibitoye, Sathish Kumar Jayapal, Jost B Jonas, Charity Ehimwenma Joshua, Himal Kandel, Getu Mosisa Kebebew, John H Kempen, Mahalaqua Nazli Khatib, Kewal Krishan, Chandrakant Lahariya, Janet L Leasher, Stephen S Lim, Roy Rillera Marzo, Andrea Maugeri, Manish Mishra, Ali H Mokdad, Admir Mulita, Christopher J L Murray, Ganesh R Naik, Zuhair S Natto, Biswa Prakash Nayak, Mohammad Negaresh, Dang H Nguyen, Bogdan Oancea, Andrew T Olagunju, Uchechukwu Levi Osuagwu, Jagadish Rao Padubidri, Songhomitra Panda-Jonas, Shahina Pardhan, Jay Patel, Arokiasamy Perianayagam, Konrad Pesudovs, Hoang Tran Pham, Elton Junio Sady Prates, Fakher Rahim, Salman Rawaf, Serge Resnikoff, Basema Saddik, Umar Saeed, Vijaya Paul Samuel, Abdallah M Samy, Tabassom Sedighi, Allen Seylani, Masood Ali Shaikh, Mohammed Shannawaz, Maryam Shayan, Aminu Shittu, Jasvinder A Singh, Yonatan Solomon, Jaimie D Steinmetz, Ian Tapply, Hugh R Taylor, Temesgen Mohammed Toma, Aristidis Tsatsakis, Guesh Mebrahtom Tsegay, Maria Viskadourou, Theo Vos, Gizachew Tadesse Wassie, and Dong Keon Yon.

### Developing methods or computational machinery

Mozhan Abdollahi, Qorinah Estiningtyas Sakilah Adnani, Hubert Amu, Mehmet Firat Baran, Rupert Bourne, Paul Svitil Briant, Kaleb Coberly, Xiaochen Dai, Thanh Chi Do, Mehdi Emamverdi, Arthur G Fernandes, Hong-Han Huynh, Sathish Kumar Jayapal, Mahalaqua Nazli Khatib, Hoda Mojiri-forushani, Ali H Mokdad, Admir Mulita, Christopher J L Murray, Hoang Tran Pham, Umar Saeed, Abdallah M Samy, Mohammed Shannawaz, Jaimie D Steinmetz, Ian Tapply, and Theo Vos.

### Providing critical feedback on methods or results

Yohannes Habtegiorgis Abate, Melsew Dagne Abate, Zahra Abbasi Dolatabadi, Mozhan Abdollahi, Richard Gyan Aboagye, Eman Abu-Gharbieh, Salahdein Aburuz, Qorinah Estiningtyas Sakilah Adnani, Shahin Aghamiri, Bright Opoku Ahinkorah, Danish Ahmad, Hamid Ahmadieh, Hooman Ahmadzadeh, Ayman Ahmed, Ahmad Samir Alfaar, Cyrus Alinia, Louay Almidani, Hubert Amu, Sofia Androudi, Jalal Arabloo, Damelash Areda, Tahira Ashraf, Sara Bagherieh, Ovidiu Constantin Baltatu, Mehmet Firat Baran, Amadou Barrow, Nebiyou Simegnew Bayileyegn, Alemshet Yirga Berhie, Jasvinder Singh Bhatti, Mukharram Bikbov, Marina G Birck, Veera R Bitra, Rupert Bourne, Marija M Bozic, Tasanee Braithwaite, Paul Svitil Briant, Alain M Bron, Katrin Burkart, Yasser Bustanji, Zahid A Butt, Robert Casson, Muthia Cenderadewi, Vijay Kumar Chattu, Omid Dadras, Xiaochen Dai, Ana Maria Dascalu, Anna Dastiridou, Vinoth Gnana Chellaiyan Devanbu, Meghnath Dhimal, Daniel Diaz, Thao Huynh Phuong Do, Thanh Chi Do, Arkadiusz Marian Dziedzic, Michael Ekholuenetale, Muhammed Elhadi, Mohammad Hassan Emamian, Mehdi Emamverdi, Hossein Farrokhpour, Arthur G Fernandes, Getahun Fetensa, Florian Fischer, Kayode Raphael Fowobaje, David S Friedman, João M Furtado, Aravind P Gandhi, Miglas W W Gebregergis, Bárbara Niegia Garcia Goulart, Mesay Dechasa Gudeta, Sapna Gupta, Vivek Kumar Gupta, Veer Bala Gupta, Golnaz Heidari, Sung Hwi Hong, Hong-Han Huynh, Segun Emmanuel Ibitoye, Irena M Ilic, Mustapha Immurana, Sathish Kumar Jayapal, Jost B Jonas, Nitin Joseph, Charity Ehimwenma Joshua, Rim Kahloun, Himal Kandel, Ibraheem M Karaye, Getu Mosisa Kebebew, John H Kempen, Mahmoud Tawfik KhalafAllah, Sudarshan Khanal, Mahalaqua Nazli Khatib, Kewal Krishan, Chandrakant Lahariya, Janet L Leasher, Stephen S Lim, Roy Rillera Marzo, Andrea Maugeri, Yang Meng, Tomislav Mestrovic, Manish Mishra, Nouh Saad Mohamed, Ali H Mokdad, Hamed Momeni-Moghaddam, Fateme Montazeri, Admir Mulita, Christopher J L Murray, Mahdi Nabi Foodani, Ganesh R Naik, Vinay Nangia, Zuhair S Natto, Biswa Prakash Nayak, Hadush Negash, Dang H Nguyen, Bogdan Oancea, Andrew T Olagunju, Matthew Idowu Olatubi, Wael M S Osman, Uchechukwu Levi Osuagwu, Jagadish Rao Padubidri, Songhomitra Panda-Jonas, Shahina Pardhan, Seoyeon Park, Jay Patel, Arokiasamy Perianayagam, Konrad Pesudovs, Hoang Tran Pham, Elton Junio Sady Prates, Ibrahim Qattea, Fakher Rahim, Mosiur Rahman, Salman Rawaf, Serge Resnikoff, Nazila Rezaei, Priyanka Roy, Basema Saddik, Umar Saeed, Sher Zaman Safi, Joseph W Sakshaug, Mohamed A Saleh, Vijaya Paul Samuel, Abdallah M Samy, Masood Ali Shaikh, Muhammad Aaqib Shamim, Mohammed Shannawaz, Bereket Beyene Shashamo, Maryam Shayan, Aminu Shittu, Emmanuel Edwar Siddig, Jasvinder A Singh, Yonatan Solomon, Jaimie D Steinmetz, Seyyed Mohammad Tabatabaei, Mohammad Tabish, Ian Tapply, Jansje Henny Vera Ticoalu, Temesgen Mohammed Toma, Guesh Mebrahtom Tsegay, Rohollah Valizadeh, Maria Viskadourou, Theo Vos, Gizachew Tadesse Wassie, Nuwan Darshana Wickramasinghe, Dong Keon Yon, and Yuyi You.

### Drafting the work or revising it critically for important intellectual content

Yohannes Habtegiorgis Abate, Melsew Dagne Abate, Mozhan Abdollahi, Eman Abu-Gharbieh, Salahdein Aburuz, Qorinah Estiningtyas Sakilah Adnani, Bright Opoku Ahinkorah, Danish Ahmad, Hamid Ahmadieh, Hooman Ahmadzadeh, Ayman Ahmed, Ahmad Samir Alfaar, Louay Almidani, Hubert Amu, Abhishek Anil, Jalal Arabloo, Alessandro Arrigo, Sara Bagherieh, Ovidiu Constantin Baltatu, Mehmet Firat Baran, Amadou Barrow, Azadeh Bashiri, Fatemeh Bazvand, Jasvinder Singh Bhatti, Mukharram Bikbov, Marina G Birck, Veera R Bitra, Rupert Bourne, Tasanee Braithwaite, Yasser Bustanji, Robert Casson, Muthia Cenderadewi, Vijay Kumar Chattu, Ana Maria Dascalu, Vinoth Gnana Chellaiyan Devanbu, Meghnath Dhimal, Daniel Diaz, Thanh Chi Do, Joshua R Ehrlich, Muhammed Elhadi, Mohammad Hassan Emamian, Mehdi Emamverdi, Arthur G Fernandes, Getahun Fetensa, Florian Fischer, Ali Forouhari, David S Friedman, João M Furtado, Miglas W W Gebregergis, Bárbara Niegia Garcia Goulart, Mesay Dechasa Gudeta, Sapna Gupta, Vivek Kumar Gupta, Veer Bala Gupta, Golnaz Heidari, Sung Hwi Hong, Hong-Han Huynh, Segun Emmanuel Ibitoye, Irena M Ilic, Mustapha Immurana, Sathish Kumar Jayapal, Jost B Jonas, Nitin Joseph, Himal Kandel, Hengameh Kasraei, Getu Mosisa Kebebew, John H Kempen, Mahmoud Tawfik KhalafAllah, Sudarshan Khanal, Mahalaqua Nazli Khatib, Kewal Krishan, Chandrakant Lahariya, Janet L Leasher, Roy Rillera Marzo, Andrea Maugeri, Tomislav Mestrovic, Hoda Mojiri-forushani, Ali H Mokdad, Hamed Momeni-Moghaddam, Fateme Montazeri, Christopher J L Murray, Vinay Nangia, Zuhair S Natto, Biswa Prakash Nayak, Mohammad Negaresh, Hadush Negash, Dang H Nguyen, Bogdan Oancea, Andrew T Olagunju, Matthew Idowu Olatubi, Wael M S Osman, Uchechukwu Levi Osuagwu, Jagadish Rao Padubidri, Songhomitra Panda-Jonas, Shahina Pardhan, Seoyeon Park, Jay Patel, Konrad Pesudovs, Hoang Tran Pham, Elton Junio Sady Prates, Ibrahim Qattea, Fakher Rahim, Deepthi Rapaka, Salman Rawaf, Nazila Rezaei, Priyanka Roy, Basema Saddik, Umar Saeed, Sare Safi, Vijaya Paul Samuel, Abdallah M Samy, Aswini Saravanan, Allen Seylani, Muhammad Aaqib Shamim, Mohammed Shannawaz, Bereket Beyene Shashamo, Aminu Shittu, Emmanuel Edwar Siddig, Jasvinder A Singh, Yonatan Solomon, Raúl A R C Sousa, Hugh R Taylor, Jansje Henny Vera Ticoalu, Temesgen Mohammed Toma, Aristidis Tsatsakis, Guesh Mebrahtom Tsegay, Maria Viskadourou, Theo Vos, Nuwan Darshana Wickramasinghe, and Dong Keon Yon.

### Managing the estimation or publications process

Rupert Bourne, Thanh Chi Do, Arthur G Fernandes, Hong-Han Huynh, Mahalaqua Nazli Khatib, Chandrakant Lahariya, Ali H Mokdad, Christopher J L Murray, Konrad Pesudovs, Hoang Tran Pham, Abdallah M Samy, and Theo Vos.

Vision Loss Expert Group of the Global Burden of Disease Study

### Providing data or critical feedback on data sources

Alessandro Arrigo, Mukharram M Bikbov, Rupert R A Bourne, Tasanee Braithwaite, Alain Bron, Ching-Yu Cheng, Maria Vittoria Cicinelli, Monte A Del Monte, Joshua R Ehrlich, Arthur Fernandes, Seth Flaxman, David Friedman, João M Furtado, Gus Gazzard, Ronnie George, M Elizabeth Hartnett, Jost B Jonas, Rim Kahloun, John H Kempen, Monef Khairallah, Rohit C Khanna, Judy E Kim, Van Charles Lansingh, Janet Leasher, Nicolas Leveziel, Kovin S Naidoo, Vinay Nangia, Michal Nowak, Konrad Pesudovs, Tunde Peto, Pradeep Ramulu, Serge Resnikoff, Tabassom Sedighi, Ian Tapply, Hugh Taylor, Fotis Topouzis, Miltiadis Tsilimbaris, Ya Xing Wang, Ningli Wang

### Developing methods or computational machinery

Rupert R A Bourne, Jost B Jonas, Ian Tapply

### Providing critical feedback on methods or results

Alessandro Arrigo, Mukharram M Bikbov, Rupert R A Bourne, Tasanee Braithwaite, Monte A Del Monte, David Friedman, João M Furtado, M Elizabeth Hartnett, Jost B Jonas, Rim Kahloun, John H Kempen, Vinay Nangia, Konrad Pesudovs, Serge Resnikoff, Ian Tapply, Ningli Wang

### Drafting the work or revising it critically for important intellectual content

Alessandro Arrigo, Mukharram M Bikbov, Rupert R A Bourne, Tasanee Braithwaite, Monte A Del Monte, Gus Gazzard, Ronnie George, M Elizabeth Hartnett, Jost B Jonas, Janet Leasher, Konrad Pesudovs

### Managing the estimation or publications process

Rupert R A Bourne, Jost B Jonas
